# Chinese physician perceptions regarding industry support of continuing medical education programs: a cross-sectional survey

**DOI:** 10.1080/10872981.2019.1694308

**Published:** 2019-11-20

**Authors:** Christopher R. Stephenson, Qi Qian, Paul S. Mueller, Cathy D. Schleck, Jayawant N. Mandrekar, Thomas J. Beckman, Christopher M. Wittich

**Affiliations:** a Division of General Internal Medicine, Mayo Clinic, Rochester, MN, USA; b Division of Nephrology and Hypertension, Mayo Clinic, Rochester, MN, USA; c Division of Biomedical Statistics and Informatics, Mayo Clinic, Rochester, MN, USA

**Keywords:** Continuing medical education, continuous professional development, industry funding, China, international, ethics

## Abstract

**Background**: Industry funding in continuing medical education has been extensively studied in the USA. Although continuing medical education is also a requirement for Chinese physicians, little is known about Chinese physician perceptions of industry support in continuing medical education.

**Objective**: We aim to determine perceptions regarding industry support for CME among Chinese physicians at a large CME course, examine potential associations between Chinese physicians’ perceptions and their demographic characteristics, and compare Chinese and US physicians’ perceptions of industry support for CME.

**Design**: We performed a cross-sectional survey of physicians at a nephrology continuing medical education conference in China. All participants received a previously published, anonymous survey consisting of 4 items, with questions asked in English and Mandarin Chinese. Responses were compared with those of a previous cohort in the USA.

**Results**: The response rate was 24% (128/541). Most respondents were nephrologists (112/126, 89%), women (91/128, 71%), and aged 20 to 40 years (79/127, 62%). Most respondents preferred industry-supported continuing medical education (84/123, 68%) or had no preference (33/123, 27%). More clinicians than clinical researchers supported industry offsetting costs (76.9% vs 58.3%; *P* = .03). Almost half of participants (58/125, 46%) stated that industry-supported continuing medical education was biased in support of industry. Compared with US physicians, Chinese physicians were more likely to believe, or had no opinion, that industry-supported courses were biased (67.2% vs 47.0%; *P* < .001).

**Conclusions**: Chinese continuing medical education participants preferred industry-sponsored continuing medical education and were strongly in favor of industry offsetting costs, but almost half believed that such education was biased in favor of supporting companies. Concern for bias was higher among Chinese than US physicians. Given participants’ concerns, further study examining industry bias in Chinese continuing medical education is recommended.

**Abbreviations**: CME: Continuing medical education; US: USA

## Introduction

Professional development, defined as ‘activities intended to improve professional knowledge, skills, or performance,’ is an expectation of all physicians in practice [1]. Continuing medical education (CME) has been described as ‘a subset of professional development that awards formal credit for completing professional development activities’ [1–5]. Formal CME requirements have been commonplace for many years in North America. Professional development requirements for physicians in the USA (US) have evolved with time to now include maintenance of certification that encompasses professional standing, lifelong learning and self-assessment, assessment of knowledge, and participation in medical practice improvement [6]. Conversely, in China, CME requirements for its 2 million physicians have been implemented in just the past 2 decades [7]. Currently, a national mandate requires Chinese physicians to have 25 hours of CME credits per year [7]. Despite the magnitude and importance of keeping Chinese physicians in practice up to date, there has been little study of CME programs in China.

Large-group, lecture-based courses remain one of the most common methods for CME [8–11]. These traditional CME courses are often held in large, conference-style sessions, primarily because it is a cost-effective method to provide continuing education [12]. However, even large, didactic-style conferences can be costly for providers. Industry support of CME is one way to offset costs. From 1998 to 2007 in the US, industry support of CME increased from $310 million to $1.2 billion per year [13]. Although this number has decreased in recent years, in 2015, 17% of all US physicians attended an accredited activity that received commercial support [9].

Critics of industry-supported CME have voiced concern that course content and presenters may be biased in favor of commercial interests [14–19]. Past research suggests that industry support in general can change physician behavior, including prescribing practices [20]. For example, physicians who received support from industry were more likely to prescribe brand-name medications [21]. Even physicians who received small gifts, such as a meal, were more likely to prescribe a more expensive brand-name medication than a cheaper generic drug [22]. Given the concern that industry support can influence physician behavior, there has been a push to regulate industry influences in CME [23]. However, proponents of industry support argue that without it, CME could become prohibitively expensive for participants [24,25]. In China, most CME funding is from industry and is intended to be bias free, although some reports have noted industry corruption in Chinese CME [7].

Given the concern for potential commercial bias, US physicians’ perceptions of industry-supported CME have been studied [25,26]. In one prior study, US physicians demonstrated no preference for courses supported by industry or not [26]. In that study, most physicians believed that industry support should be used to offset costs but did not believe that industry-sponsored CME was biased. Preference for industry support of CME programs varied by time in practice – physicians in practice for more than 30 years more often preferred industry-supported courses and those with exhibitors [26]. A more recent 2011 study did demonstrate that US physicians perceived industry-supported CME as more biased, but physicians did not support the removal of industry support to offset costs [25].

In contrast to US physicians, little is known about Chinese physicians’ perceptions of industry support for CME programs. To fill this gap, we conducted a cross-sectional study at a large CME course attended by Chinese physicians. The aims of this study were to 1) determine perceptions regarding industry support for CME among Chinese physicians at a large CME course, 2) examine potential associations between Chinese physicians’ perceptions and their demographic characteristics, and 3) compare Chinese and US physicians’ perceptions of industry support for CME [26]. We hypothesized that Chinese physician perceptions of industry support for CME would be similar to those of physicians in the US, with similar concerns for bias in industry-supported CME.

## Materials and methods

### Participants and setting

We performed a cross-sectional survey study of all attendees at Nephrology Update West Lake Forum in Hangzhou, China, in 2017 [27]. The Nephrology Update West Lake Forum is an annual 5-day course that is jointly organized by the Mayo Clinic Alix School of Medicine, Second Military Medical University, and China and Zhejiang University. The course provides nephrologists, internists, and other practicing health care providers with clinically relevant updates for kidney disease and related conditions. The course consists of podium presentations and workshops. Course faculty are chosen by the course directors and are experts in their field. The course offers 20 CME credits and is endorsed by the American Society of Nephrology and the International Society of Nephrology. The conference received industry support to offset the costs of food and participant transportation to and from the course. Industry groups also could buy space and set up displays in an exhibit hall. This study was deemed exempt by the Mayo Clinic and Zhejiang University institutional review boards.

### Data collection

At the conference, a survey packet was distributed to each attendee at the time of registration for the course. The survey packet content included demographic questions and the survey regarding industry funding perceptions. The 4 previously reported questions [26] regarding industry support of CME activities were 1) What type of CME course do you prefer to attend? (industry supported, non–industry supported, no preference); 2) Do you prefer a CME course with or without exhibitors? (with exhibitors, without exhibitors, no preference); 3) Do you believe CME courses should accept industry support if doing so reduces the overall cost of the course? (yes, no, no preference); and 4) Is it your impression that the contents of CME courses supported by industry tend to be biased in favor of the supporting companies? (yes, no, no opinion). The survey packets were translated by a professional language service and printed in both English and Mandarin Chinese. At the end of the conference, participants returned their completed evaluation packets to the course registration desk and received a pen for participation. The Mayo Clinic survey research center compiled the results using double entry to ensure data accuracy.

### Historical comparison with US CME participants

Responses from Chinese participants were compared with those from a previously studied US CME population using this same survey with a similar methodology [26]. The previous study was a cross-sectional, anonymous survey of physicians attending 4 Mayo Clinic internal medicine CME courses conducted in 2004. Respondents at these previous courses completed their survey packet in person during a break period in the middle of each course. The corresponding author of the 2004 study provided the data from that study to assist with data interpretation and compare the 2004 cohort with the Chinese cohort.

### Data analysis

Categorical variables are presented as number (percentage). Associations between participants’ demographic variables, the survey item responses, and the historical comparisons were determined using the Fisher exact test. Statistical significance was set at *P* < .05. Statistical analyses were conducted using SAS version 9.4 (SAS Institute Inc).

## Results

### Participant characteristics

Of the 541 surveys distributed, 128 surveys were returned (response rate, 24%). Demographic characteristics of the Chinese respondents are shown in Table 1. All physicians who responded to the survey were practicing physicians in China. Most respondents were nephrologists (112/126, 89%), women (91/128, 71%), and aged 20 to 40 years (79/127, 62%). Most respondents spent 31 to 50 hours per week providing patient care (68/127, 54%). A large proportion of participants reported being in practice for only 0 to 10 years (57/127, 45%) and had no academic rank (50/125, 40%).

**Table 1.** Characteristics of survey respondents

Characteristic	No. of Respondents (%) (N = 128)
Age, y	(n = 127)
20–30	39 (30.7)
31–40	40 (31.5)
41–50	28 (22.0)
51 or older	20 (15.7)
Gender	
Male	37 (28.9)
Female	91 (71.1)
Specialty	(n = 126)
Nephrology	112 (88.9)
Internal medicine	10 (7.9)
Other	4 (3.2)
Primary position	(n = 123)
Clinician	111 (90.2)
Researcher	12 (9.8)
Time in clinical practice, y	(n = 127)
0–10	57 (44.9)
11–20	27 (21.3)
21–30	29 (22.8)
31 or more	14 (11)
Academic rank	(n = 125)
None	50 (40.0)
Instructor	35 (28)
Assistant Professor/Associate Professor	22 (17.6)
Professor	18 (14.4)

The previously studied US comparison group had a total of 1,130 responses (response rate, 70.5%) and was primarily older men (77.4% men; 53.6% older than 50 years) [26].

### Perceptions of industry support

Among 123 respondents to question 1, most preferred industry-supported CME (84, 68%) or had no preference (33, 27%) (Table 2). Very few participants (6, 5%) preferred a non–industry-supported CME course. For question 2, most of the 124 respondents expressed no preference whether exhibitors were present at their CME course (69, 56%) (Table 2). For the 55 who had a preference, most preferred exhibitors (39, 71%) compared with a CME course without exhibitors.

**Table 2.** Responses to Questions 1and 2^a.^

	Question 1: What Type of CME Course Do You Prefer to Attend?				Question 2: Do You Prefer a CME Course With or Without Exhibitors?			
Respondent Category	Industry Supported (n = 84)	Non–Industry Supported (n = 6)	No Preference (n = 33)	*P*	With Exhibitors (n = 39)	Without Exhibitors (n = 16)	No Preference (n = 69)	*P*
Age, y				.53	(n = 38)			.049
20–30	30 (76.9)	1 (2.6)	8 (20.5)		15 (38.5)	1 (2.6)	23 (59.0)	
31–40	23 (60.5)	2 (5.3)	13 (34.2)		12 (30.8)	5 (12.8)	22 (56.4)	
41–50	18 (66.7)	3 (11.1)	6 (22.2)		4 (14.8)	8 (29.6)	15 (55.6)	
51 or older	13 (72.2)	0 (0.0)	5 (27.8)		7 (38.9)	2 (11.1)	9 (50.0)	
Gender				.47				.20
Male	28 (75.7)	2 (5.4)	7 (18.9)		16 (43.2)	4 (10.8)	17 (45.9)	
Female	56 (65.1)	4 (4.7)	26 (30.2)		23 (26.4)	12 (13.8)	52 (59.8)	
Primary position	(n = 80)		(n = 32)	.74			(n = 64)	.76
Clinician	72 (67.9)	5 (4.7)	29 (27.4)		36 (33.6)	14 (13.1)	57 (53.3)	
Researcher	8 (66.7)	1 (8.3)	3 (25.0)		3 (25.0)	2 (16.7)	7 (58.3)	
Academic rank	(n = 82)		(n = 31)	.07	(n = 38)		(n = 67)	.02
None	34 (70.8)	0 (0)	9 (40.9)		17 (34.7)	3 (6.1)	29 (59.2)	
Instructor	26 (76.5)	3 (8.8)	5 (14.7)		13 (38.2)	4 (11.8)	17 (50.0)	
Assistant/Associate Professor	11 (50.0)	2 (9.1)	4 (25.0)		2 (9.1)	8 (36.4)	12 (54.5)	
Professor	11 (68.8)	1 (6.3)	14 (29.2)		6 (37.5)	1 (6.3)	9 (56.3)	

^a^Values are No. of respondents (%).

Among 125 respondents to question 3, 96 expressed a preference, almost all of whom (93, 97%) reported that industry should help to reduce the costs of CME (Table 3). Approximately one-fourth of respondents (29, 23%) did not state a preference. For 125 respondents to question 4, 99 expressed an opinion, most of whom (58, 56%) stated that industry-supported courses were biased (Table 3). One-fifth (26, 21%) had no opinion. Among all respondents, about half (58, 46%) were concerned about industry-supported biases.

**Table 3.** Responses to Questions 3 and 4^a.^

	Question 3: Should CME Courses Accept Industry Support?				Question 4: Are Industry-Supported Courses Biased?			
Respondent Category	Yes (n = 93)	No (n = 3)	No Preference (n = 29)	*P*	Yes (n = 58)	No (n = 41)	No Opinion (n = 26)	*P*
Age, y	(n = 92)			.89	(n = 57)			.17
20–30	30 (76.9)	1 (2.6)	8 (20.5)		15 (38.5)	15 (38.5)	9 (23.1)	
31–40	29 (74.4)	2 (5.1)	8 (20.5)		22 (56.4)	11 (28.2)	6 (15.4)	
41–50	20 (74.1)	0 (0.0)	7 (25.9)		15 (55.6)	5 (18.5)	7 (25.9)	
51 or older	13 (68.4)	0 (0.0)	6 (31.6)		5 (26.3)	10 (52.6)	4 (21.1)	
Gender				.30				.90
Male	28 (75.7)	2 (5.4)	7 (18.9)		18 (48.6)	11 (29.7)	8 (21.6)	
Female	65 (73.9)	1 (1.1)	22 (25.0)		40 (45.5)	30 (34.1)	18 (20.5)	
Primary position	(n = 90)		(n = 27)	.03	(n = 57)	(n = 39)	(n = 24)	.57
Clinician	83 (76.9)	1 (0.9)	24 (22.2)		53 (49.1)	34 (31.5)	21 (19.4)	
Researcher	7 (58.3)	2 (16.7)	3 (25.0)		4 (33.3)	5 (41.7)	3 (25.0)	
Academic rank			(n = 27)	.29	(n = 56)	(n = 40)		.46
None	38 (77.6)	0 (0.0)	11 (22.4)		17 (34.7)	20 (40.8)	12 (24.5)	
Instructor	26 (76.5)	3 (8.8)	5 (14.7)		20 (58.8)	9 (26.5)	5 (14.7)	
Assistant/Associate Professor	16 (72.7)	0 (0.0)	6 (27.3)		10 (45.5)	6 (27.3)	6 (27.3)	
Professor	12 (70.6)	0 (0.0)	5 (29.4)		9 (52.9)	5 (29.4)	3 (17.6)	

^a^Values are No. of respondents (%).

### Associations between participant characteristics and perceptions of industry support

Participant characteristics, including age, gender, primary position, and academic rank, had sufficient data in each category to determine associations with responses to the survey and were included in the associations analysis. For question 1, preference for course type, and question 4, bias in industry-supported courses, there were no significant associations between preferences the participants’ age, gender, primary position, specialty, or academic rank, with all ages and academic ranks preferring industry-supported courses. For question 2, there were significant differences by age (*P* = .049) and academic rank (*P* = .02), with assistant professors and those aged 41 to 50 years less likely to prefer exhibitors. For question 3, clinicians were more likely than clinical researchers to support industry offsetting costs (76.9% vs 58.3%; *P* = .03). There was no significant difference between preference and age or academic rank.

### Comparison with historical US CME cohort

**Figure 1.**
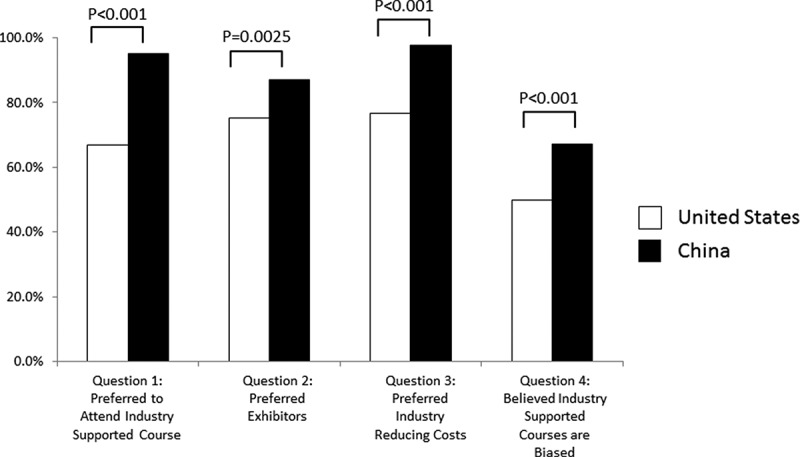
Attitudes on industry funding in continuing medical education among chinese and USA Physicians

Conceptually, a response of ‘no preference’ was thought to represent indifference to industry funding, rather than opposition to funding. Given this, in the cohort comparisons, ‘yes’ and ‘no preference/opinion’ responses were combined to indicate preference or indifference for industry support. Compared with a previously studied population in the US, Chinese participants had more favorable views of industry-supported CME (95.1% vs 66.7%; *P* < .001) (Figure 1). Similarly, Chinese physicians preferred, or had no preference for, exhibitors (87.1% vs 75.2%; *P* = .003) and preferred, or had no preference for, industry reducing costs (97.6% vs 76.5%; *P* < .001). Chinese physicians were also more likely to believe, or had no opinion, that industry-supported courses were biased (67.2% vs 47.0%; *P* < .001).

## Discussion

### Perceptions of industry support

To our knowledge, this is the first study to evaluate perceptions of industry support in Chinese CME. Overall, Chinese CME participants preferred industry-supported CME and were strongly in favor of industry offsetting costs, regardless of age, academic rank, or practice type. The majority of Chinese physicians surveyed preferred to attend industry-supported courses, had no preference for the inclusion of exhibitors, and believed that industry should offset the costs of CME. Interestingly, even though Chinese physicians supported industry involvement in CME, almost half of participants believed that industry support created bias in CME. Using industry to offset costs may be especially needed in China, which is still considered a developing country. Offsetting costs may be required for participants to afford to travel and attend, although participants may still be skeptical of the information presented.

Interestingly, the preference for industry support was fairly consistent despite participant age, gender, academic rank, or practice type. Several significant differences were noted regarding preference for exhibitors and preference for industry reducing costs. However, these differences are challenging to interpret given the low numbers of responses for these demographics. These could represent a type I error given the number of demographics collected, although more research into these demographics and other practice types should be considered.

### Integration with prior research

The majority of both US and Chinese physicians preferred industry-supported CME. However, when comparing the Chinese cohort with the historical US cohort, Chinese physicians were more likely to prefer industry-supported CME, although the concern for bias was higher in Chinese physicians [26]. Interestingly, these observations suggest that Chinse physicians may be more skeptical of industry, even though they preferred industry-supported CME courses overall. This result is likely multifactorial and may stem from cultural differences between physicians living in a predominantly communist country, such as China, where the population may be more guarded toward industry, and US physicians living in a capitalist country, who may have more positive views toward industry and fewer concerns of bias. Indeed, this aligns with prior studies suggesting that Chinese nationals may hold ideologies shaped by Marxism and Confucianism, leading to more anti-capitalist worldviews [28]. Furthermore, Chinese skepticism toward industry in CME may also be secondary to several recent scandals where foreign pharmaceutical companies were charged with bribing physicians, hospitals and government employees [7].

In the US cohort, younger physicians were less likely than older physicians to prefer industry support [26], which was not seen in our study. The Chinese cohort was, on average, younger than the US cohort and still expressed a strong preference for industry-supported CME. Chinese physicians are often poorly compensated and, despite skepticism toward industry, Chinese physicians may require industry-supported financial assistance for travel expenses and to attend costly CME meetings [29].

### Study implications

The study’s implications yield several lessons learned. First, China has one of the largest physician populations in the world, and ensuring that these physicians remain competent is a health care priority for China. However, China is also a developing country, and travel, conference registration, and lodging costs may present a barrier to attending high-quality CME courses. By offsetting costs, industry support may allow access to high-quality CME programs for Chinese physicians who otherwise could not afford to attend. Second, industry should also be aware that Chinese physicians may be more skeptical of information presented. Third, given the skeptical nature of Chinese CME attendees, it is imperative that CME conferences use best practices when delivering information, including resolving personal conflicts of interests, disclosing financial relationships, and presenting content without commercial bias [30].

### Limitations and strengths

This study has several limitations. First, our 24% response rate was low, which could contribute to a selection bias. Second, our population consisted primarily of young nephrologists, which may limit the generalizability of our findings. Even though we did not see a difference on the basis of years in practice, age, or academic rank, this could be related to a type II error because most of our respondents were younger physicians, with fewer experienced physicians in our sample. Conversely, compared with the US cohort, we would have expected younger physicians to be less supportive of industry-supported CME, which we did not observe. Last, perceptions of CME in the US may have changed from 2004 to 2017. Indeed, a more recent study from 2011 does show that US physicians may now perceive industry-supported CME as more biased, but a different instrument was used to assess perceptions in the 2011 study [25].

Our study also has several strengths. First, we used a previously described survey on industry support in CME and applied it to a population living in a communist country, which, to our knowledge, has not been done before. Additionally, we linked the participants’ demographics and characteristics to their survey responses. To assist with our study’s generalizability, we also selected a large didactic CME conference, which is the traditional method in which CME is delivered. We also compared our study results with a historical US CME cohort to identify differences between physician perceptions in 2 countries.

Further research into industry funding perceptions of CME should be considered. First, given the changing nature of CME in the USA with integration of Maintenance of Certification, it would be reasonable to restudy US physicians to determine whether their perceptions of industry support have changed. Additionally, future study of industry funding in CME in other countries with different economic and market systems should be considered to see if these perceptions support the findings of our study.

### Conclusion

Overall, in a sample of Chinese nephrologists attending a CME conference, there is strong support for industry support of CME despite concerns about industry bias. Given these preferences, further study examining industry perceptions in other Chinese CME populations is recommended.

## Data Availability

The dataset supporting the conclusions of this article can be found at the following link: https://www.dropbox.com/s/34dy71db0tm8doc/Dataset.xlsx?dl=0
